# Optical Properties of Phenylthiolate-Capped CdS Nanoparticles

**DOI:** 10.1021/acs.jpcc.4c06753

**Published:** 2025-01-10

**Authors:** Eimear Madden, Martijn A. Zwijnenburg

**Affiliations:** Department of Chemistry, University College London, 20 Gordon Street, London WC1H 0AJ, U.K.

## Abstract

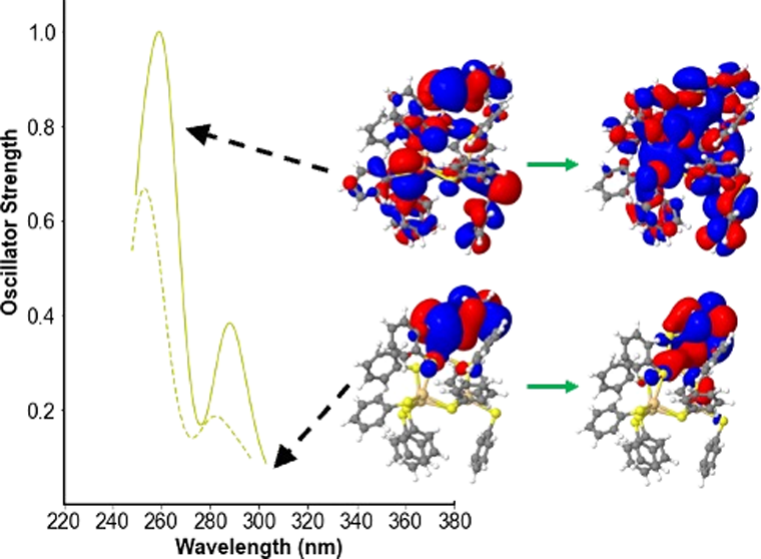

Using many-body perturbation theory, we study the optical properties of phenylthiolate-capped cadmium sulfide nanoparticles to understand the origin of the experimentally observed blue shift in those properties with decreasing particle size. We show that the absorption spectra predicted by many-body perturbation theory agree well with the experimentally measured spectra. The results of our calculations demonstrate that all low-energy excited states correspond to a mixture of two fundamental types of excitations: intraligand and ligand-to-metal charge-transfer excitations. We find that for each excited state, the intraligand excitation contribution is dominant and that bright excited states, corresponding to the clear peaks in the absorption spectra, have a larger ligand-to-metal charge-transfer contribution. There are no low-energy bulk-like excitons, excited states for which both the hole and the excited electron components are predominantly delocalized over the inorganic core of the particles. Phenylthiolate-capped cadmium sulfide nanoparticles appear not to behave like the textbook cartoon picture of quantum dots. We speculate that the observed blue shift is the result of a combination of a Stark-like shift of the intraligand contribution, modulated by a change in the charge of the inorganic core and the confinement of the excited electron component of the ligand-to-metal charge-transfer contribution.

## Introduction

**Figure 1:**
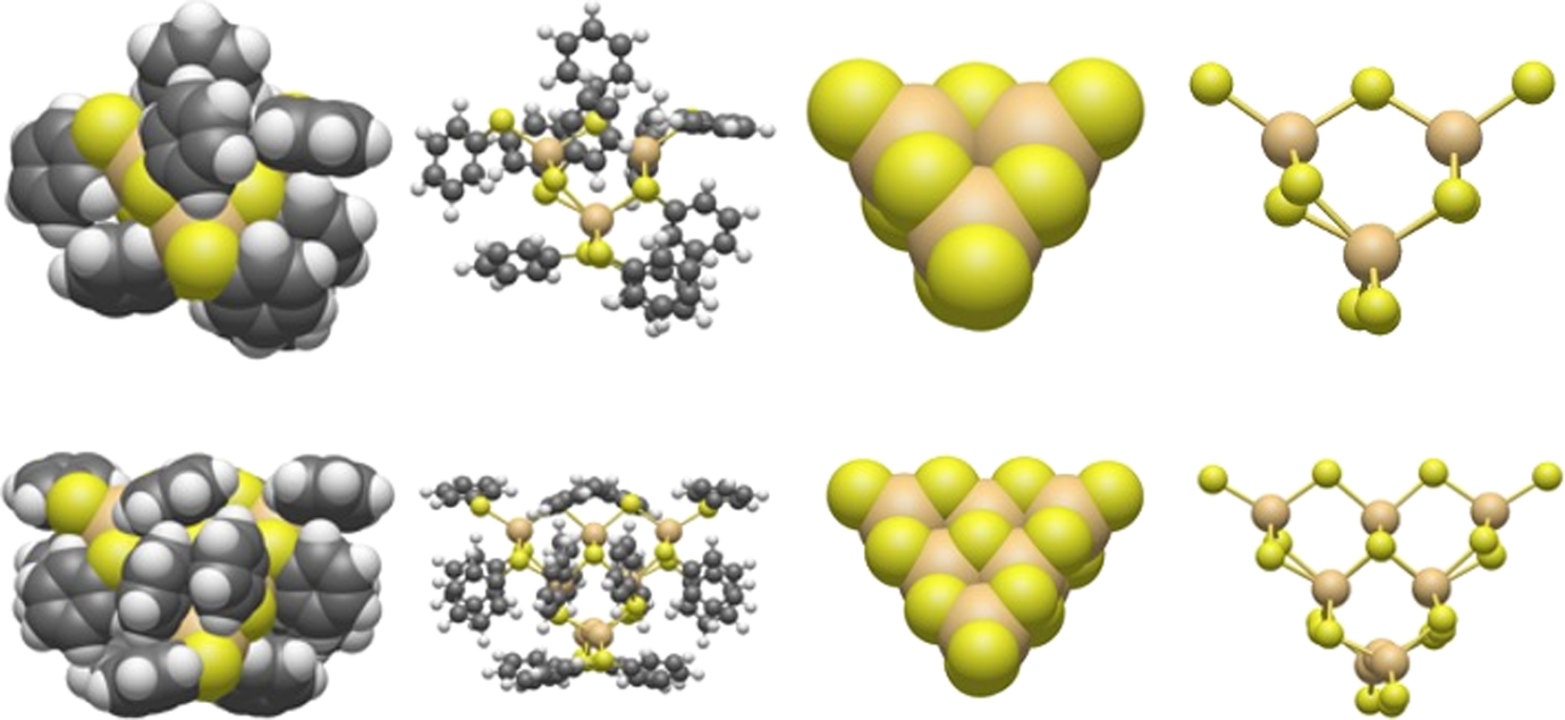
Pictures of the DFT-optimized structures of the [Cd_4_(SPh)_10_]^2–^ (top row) and [Cd_10_S_4_(SPh)_16_]^4–^ (bottom row) nanoparticles, showing for each particle from left to right, the particle including ligands with the atoms rendered as van der Waals spheres, the particle including ligands shown in a ball-and-stick representation, the inorganic core of the particle rendered as van der Waals spheres, and the inorganic core of the particle shown in a ball-and-stick representation. Cadmium atoms are shown in gold, sulfur atoms in yellow, carbon atoms in gray, and hydrogen atoms in white.

Phenylthiolate-capped cadmium sulfide nanoparticles are among the most structurally well-defined semiconductor nanomaterials. Atomically precise [Cd_4_(SPh)_10_]^2–^ and [Cd_10_S_4_(SPh)_16_]^4–^ nanoparticles with a zincblende CdS core (see Figure 1), and where the S^2–^ sulfide anions on the corners and edges of the core have been replaced with PhS^–^ phenylthiolate anions, have been crystallized as salts with alkylammonium counterions and their structure solved from X-ray diffraction.^1,2^ Larger CdS zincblende particles capped with phenylthiolate with core sizes of up to approximately 30–35 nm and seemingly narrow particle size distribution were also reported in the literature.^3^ For both the atomically precise nanoparticles and the (larger) nanoparticles, the onset of light absorption, the optical gap, and fluorescence maximum blue-shift with decreasing particle size,^3,4^ as commonly observed for semiconductor nanoparticles.^5^

Due to their structurally well-defined nature and the availability of experimental spectroscopic data for (atomically precise) phenylthiolate, capped CdS particles are an interesting model system for nanoparticles of compound semiconductors in general. We can then use phenylthiolate-capped CdS particles to consider questions such as (i) how closely do excited states in such relatively nonuniform particles with complex ligands resemble the textbook cartoon picture of quantum confinement, where a bulk-like excitation is “clamped” by the size of the particle, resulting in a blue shift with decreasing particle size, and (ii) does a blue shift with decreasing particle size necessarily imply quantum confinement?

Wang and Herron^6^ showed that effective mass calculations qualitatively describe the trend in the experimentally observed optical gap, and that tight-binding calculations quantitatively describe the optical gap data for particles larger than 20 nm with a deviation in the case of small particles. Both the effective mass and tight-binding model assume that the relevant excitations in the nanoparticle are bulk-like and do not involve the ligands. The same authors in another report^3^ observe a dependency of the fluorescence intensity on the phenylthiolate-to-sulfide ratio for the particles, which they, in contrast to the absorption spectra, interpret to indicate that the fluorescent state involves the phenylthiolate ligands. Tuerk and co-workers^4^ propose that the absorption spectra of the particles are a mixture of intraligand and ligand-to-metal charge-transfer excitations and that the fluorescence is due to a ligand-to-metal charge-transfer excitation.

The picture from the experiment is clearly complicated. Hence, ab initio calculations that do not presuppose a particular mechanism or (de)localization of the relevant excited states (in contrast to the above-mentioned effective mass and tight-binding calculations) are required to unequivocally answer questions about the character of the excited states in phenylthiolate-capped CdS particles and the origin of the blue shift.

Density functional theory, DFT, and its time-dependent extension, time-dependent DFT, TD-DFT, would be the natural framework to perform ab initio calculations on nanoparticles, owing to their favorable scaling with system size. However, with practical exchange–correlation functionals, TD-DFT struggles with the description of charge-transfer states,^7,8^ such as the above-mentioned ligand-to-metal charge-transfer states. TD-DFT calculations can reproduce experimental absorption spectra, for example, for magnesium oxide nanoparticles,^9^ or the results of high-level quantum chemistry calculations, for example, in the case of titanium dioxide clusters.^10,11^ However, for each system, even in the absence of ligands, this requires a careful selection of the exchange–correlation functional in general, and tuning the amount of exact exchange in the functional used in particular, to minimize issues with charge-transfer states, making such calculations much more empirical than desired. High-level quantum chemistry calculations, such as equation-of-motion coupled-cluster calculations, do not suffer from the drawbacks of TD-DFT but do not scale sufficiently favorably with system size to make calculations on phenylthiolate-capped nanoparticles computationally tractable. *GW*-BSE many-body perturbation theory calculations based on Green’s functions on top of DFT, where *GW*(12−14) is used to calculate the quasiparticle spectrum and the Bethe–Salpeter equation^15−17^ is solved using the predicted quasiparticle spectrum as input to predict the exciton or optical absorption spectrum, similarly does not suffer from the drawbacks of TD-DFT. However, in contrast to high-level quantum chemistry methods, *GW*-BSE scales sufficiently favorably with system size, even if still much more computationally expensive than TD-DFT, such that calculations on phenylthiolate-capped nanoparticles with *GW*-BSE are computationally tractable.

Previously, the optical properties of [Cd_4_(SH)_10_]^2–^ and [Cd_10_S_4_(SH)_16_]^4–^, where the phenylthiolate ligands have been replaced by bisulfide (SH^–^) ions, and related anionic particles were studied using tight-binding TD-DFT by Frenzel et al.^18^ Zhu and co-workers^19^ used *GW*-BSE to predict the optical properties of the sodium salts of anionic bisulfide-capped particles with CdS cores with a so-called mixed structure, an intergrowth of the zincblende and wurtzite structure. Lopez del Peurto^20^ studied the properties of closely related tetrahedral CdSe structures capped with fictitious atoms instead of ligands by *GW*-BSE calculations, while we previously used *GW*-BSE to study cubic CdS nanoparticles cut from the rocksalt polymorph (and closely related MgO and CdO nanoparticles).^21,22^ Finally, the optical and electronic properties of hydrogenated silicon nanoparticles, the simplest elemental semiconductor analogue of the CdS particles, have been extensively studied theoretically.^23−25^ This includes a recent paper^25^ in which we used *GW*-BSE and TD-DFT to study the delocalization of excited states in these particles and showed that their behavior resembles the textbook cartoon picture of a quantum dot.

**Figure 2:**
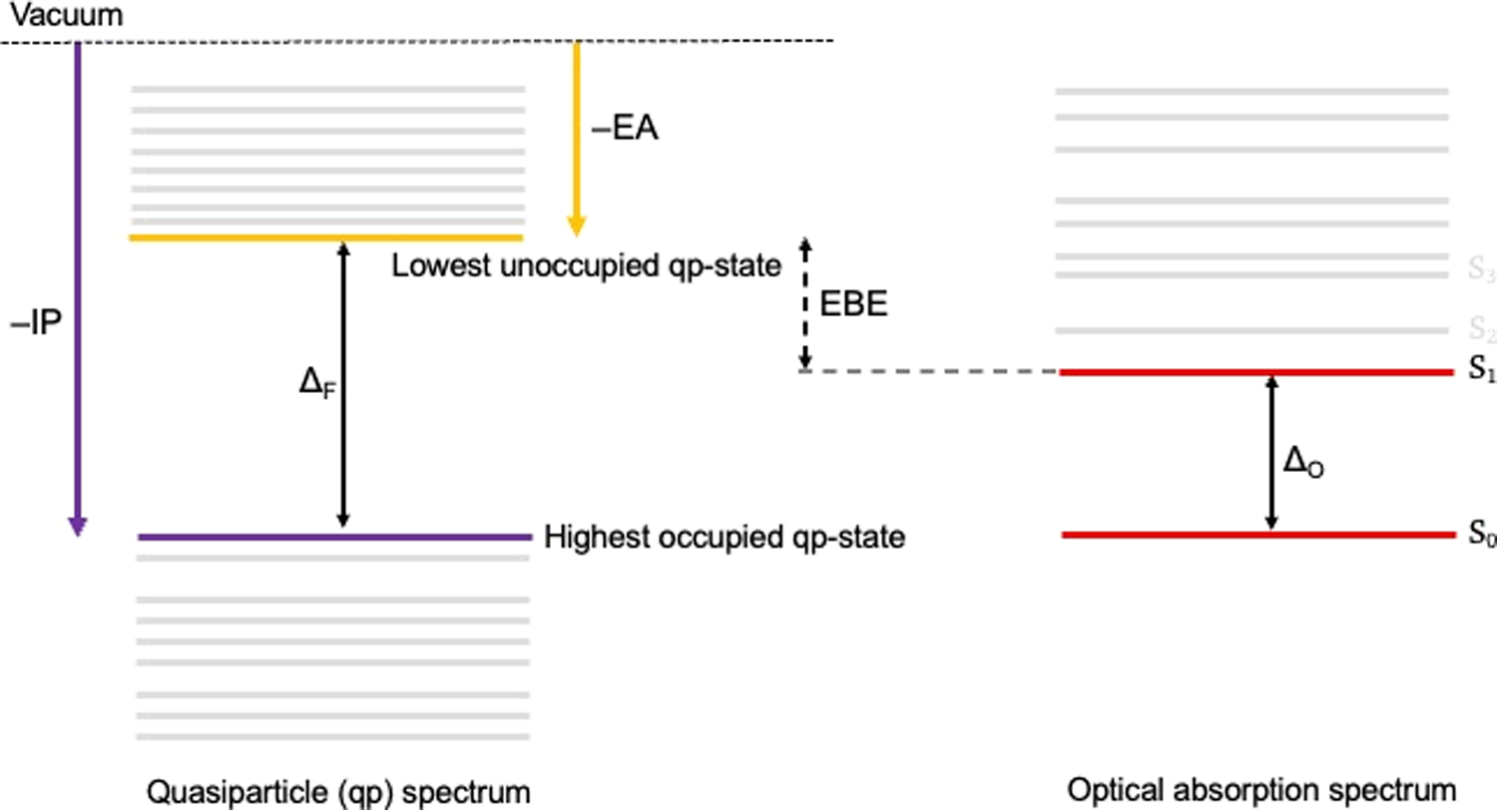
Schematic illustrating the quasiparticle and optical absorption spectra and the definition of (i) the highest occupied and lowest unoccupied quasiparticle states (−IP and −EA), (ii) the fundamental gap (Δ_F_, the energy required to generate a noninteracting electron–hole pair and the gap between the highest occupied and lowest unoccupied quasiparticle states), (iii) the optical gap (Δ_O_, the energy required to generate an interacting electron–hole pair, i.e., an exciton), and (iv) the exciton binding energy (EBE, the difference between the fundamental and optical gaps).

In this study, we use *GW*-BSE to predict the optical gap values, defined as the lowest-energy singlet-to-singlet excitation (see Figure 2), and optical absorption spectra of phenylthiolate-capped CdS particles, as well as the character of the different underlying excited states. We also consider the convergence of the predicted spectra with basis-set size and compare the predicted spectra with those measured experimentally by Tuerk and co-workers.^4^ Finally, we compare the predicted properties of the phenylthiolate-capped CdS particles with those of bulk CdS and hydrogenated silicon nanoparticles, including the predicted exciton binding energies (EBEs) (Figure 2).

## Methodology

Starting structures for the particles were taken from published experimental solid-state structures obtained via crystallography.^1,2^ The DFT geometry optimization calculations were performed using the B3LYP^26−28^ hybrid exchange–correlation functional in combination with the D4^29^ dispersion correction and the def2-SVP, def2-TZVPP, or def2-QZVPP basis-sets.^30^ For selected smaller particles, frequency calculations were performed to confirm that the stationary point found is a minimum rather than a saddle point. All of these calculations, as well as the *GW* and BSE calculations discussed below, were performed using Turbomole 7.5.^31^

The quasiparticle spectra of the particles were calculated by using *GW*, starting from the orbitals obtained from ground-state DFT calculations, which used the same B3LYP functional and basis-sets discussed above. In selected cases also, additional calculations were performed with the def2-TZVPPD or def2-QZVPPD basis-sets with additional diffuse functions.^32^ The bulk of the reported *GW* calculations are eigenvalue self-consistent *GW*, ev*GW*, calculations but we also report the result of singlet shot *GW* (*G*_0_*W*_0_) and quasiparticle self-consistent qs*GW* calculations in the Supporting Information (Tables S1 and S2), all as implemented in Turbomole.^33−35^ In the qs*GW* calculations, the self-energy is calculated using a spectral representation and in the *G*_0_*W*_0_ and ev*GW* calculations via analytical continuation. In the latter *G*_0_*W*_0_ and ev*GW* calculations, only the highest occupied and lowest unoccupied quasiparticle states are calculated explicitly using *GW*, while the remainder of the quasiparticle states are the Kohn–Sham states or more strictly the generalized Kohn–Sham states as we use a hybrid functional, shifted accordingly. Because of this approximation, the computational scaling of the *G*_0_*W*_0_ and ev*GW* implementation is much more favorable than that for qs*GW*, allowing us to study much larger particles. Our qs*GW* calculations, in contrast, were limited to the smallest systems. The lowest optical excited states were calculated by solving the Bethe–Salpeter equation on top of the *GW* quasiparticle spectrum.^36^

For *G*_0_*W*_0_, and by extension *G*_0_*W*_0_-BSE, the predicted fundamental and optical gaps will depend on the functional used in the DFT calculation the *GW* calculation is started from, here B3LYP. The use of ev*GW* and qs*GW* reduces and practically eliminates, respectively, this starting-point dependency by iterating the eigenvalues or the underlying ground state, respectively, until self-consistency is achieved. Moreover, in the case of finite-sized systems, such as the particles studied here, the results of both ev*GW*-BSE and qs*GW*-BSE agree well with coupled-cluster benchmarks, as explicitly shown for singlet excitation energies of organic molecules, and yield excitation energies there that are clearly superior to those obtained from *G*_0_*W*_0_-BSE.^33^

The character of the quasiparticle states is analyzed in terms of the underlying Kohn–Sham states. The character of the optical excited states, the excitons, is analyzed in terms of the corresponding natural transition orbitals (NTOs),^37^ as well as a series of descriptors developed by Plasser and co-workers, such as the root-mean-square electron–hole separation and the CT character.^38−41^ The latter analysis is performed using the TheoDORE code.^42^ Absorption spectra were plotted by representing all BSE excitations in a given window above the lowest excited states as Gaussian functions centered at the respective excitation energies, with a height given by the excitation’s predicted oscillator strength and a width of 0.1 eV. The specific width value was chosen such that the predicted spectra have a similar broadening as observed in the experiment.

Where relevant, the properties of the particles are all plotted relative to the radius *R* of the inorganic core of the particles. *R* is calculated from the average edge length of the inorganic cores *L* measured from the corner cadmium atoms, as the distance between the centroid of the particle and the vertices assuming the particles are ideal tetrahedra
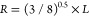
1

## Results

### Basis-Set Convergence

**Figure 3:**
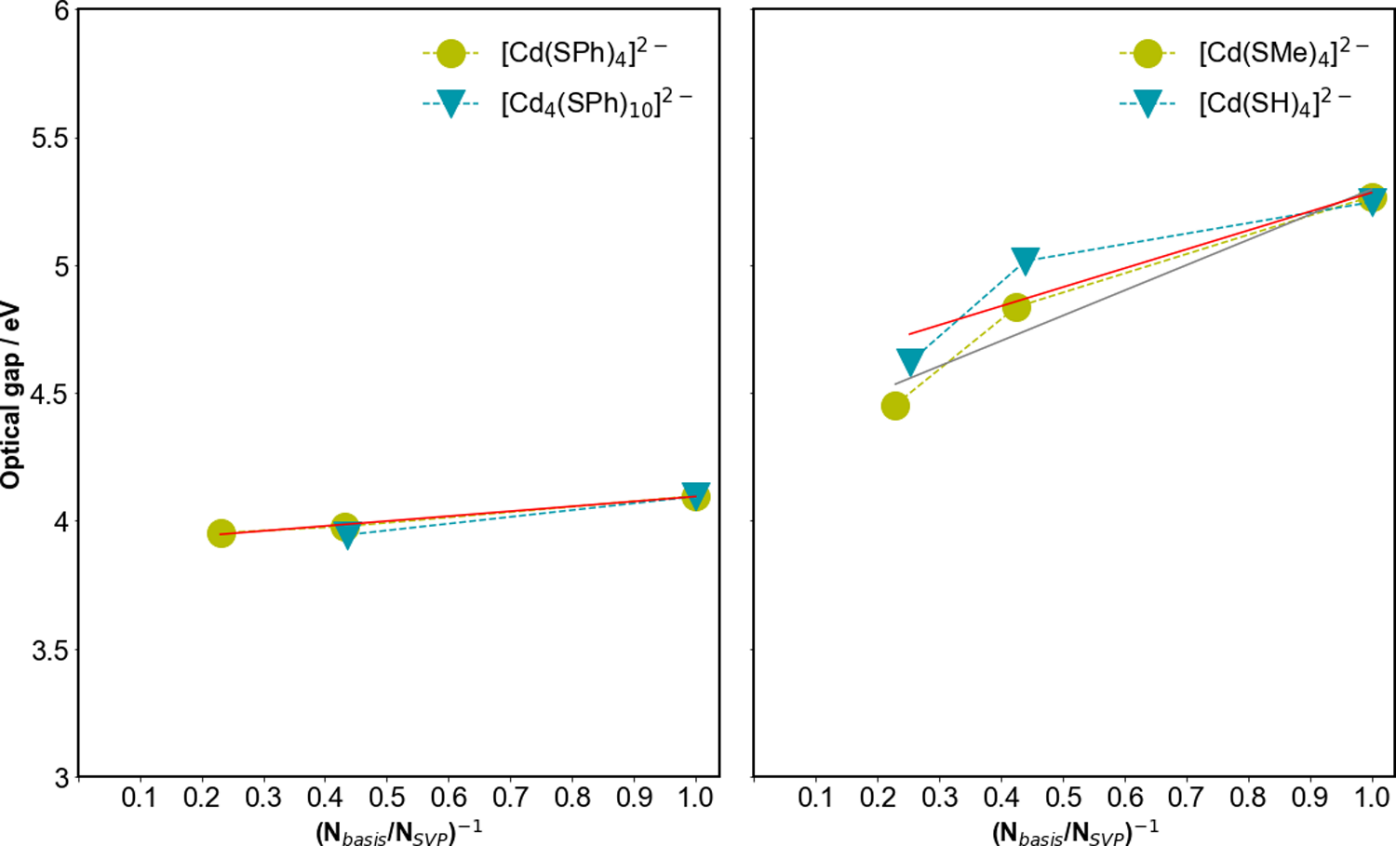
Change in the predicted optical gap with increasing basis-set size when going from def2-SVP to def2-QZVPP for phenylthiolate-capped (left) and methylthiolate- and bisulfide-capped systems (right panel). Solid lines are linear fits to the predicted optical gap values (left panel, red line fit for [Cd(SPh)_4_]^2–^; right panel, gray line fit for [Cd(SH)_4_]^2–^, and red line fit for [Cd(SMe)_4_]^2–^), while the dashed lines are there to guide the eyes.

We start by discussing the basis-set convergence of the predicted optical gap values for the particles. We study this by calculating the optical gap values with the split-valence def2-SVP, triple-ζ def2-TZVPP, and quadruple-ζ def2-QZVPP basis-sets for the [Cd_4_(SPh)_10_]^2–^ particle and the [Cd(SPh)_4_]^2–^ monomer, which are small enough to make calculations with the larger basis-sets tractable. For [Cd(SPh)_4_]^2–^, we can extrapolate the predicted optical gap to the basis-set limit by plotting the optical gap values versus one over the number of basis-functions (see Figure 3). The def2-SVP, def2-TZVPP, and def2-QZVPP optical gaps predicted for [Cd(SPh)_4_]^2–^ lie less than 0.2 eV, less than 0.1 eV, and about 0.05 eV from the extrapolated basis-set limit value (3.90 eV; see Table S3), respectively. For [Cd_4_(SPh)_10_]^2–^, the def2-QZVPP calculations proved intractable; however, the optical gap predicted using the def2-SVP and def2-TZVPP basis-sets differ by only 0.15 eV (see Table S3), which is similar in magnitude to the identical shift for [Cd(SPh)_4_]^2–^.

We also considered the change of the optical gap with basis-set for the neutral [Cd(SPh)_4_]^2–^: 2 [N(CH_3_)_4_]^+^ ion pair, which (might) exist in the solid state and solutions in a low dielectric permittivity solvent. There are potentially many geometries for such an ion pair, but we focus on just one here, which we have verified to be a minimum by calculating its harmonic frequencies. When going from def2-SVP to def2-TZVPP, the optical gap of [Cd(SPh)_4_]^2–^: 2 [N(CH_3_)_4_]^+^ shifts down by 0.15 eV (see Table S4), similar to what we see for the [Cd(SPh)_4_]^2–^ dianion.

We also explore the use of basis-sets with additional diffuse functions in the form of the def2-TZVPPD and def2-QZVPPD basis-sets. In this case, for [Cd(SPh)_4_]^2–^, the optical gap shifts rigidly downward by 0.6 eV relative to the def2-TZVPP and def2-QZVPP results (see Table S5). Similarly, for the [Cd(SPh)_4_]^2–^: 2 [N(CH_3_)_4_]^+^ ion pair, the def2-TZVPPD optical gap is shifted downward by 0.3 eV relative to its def2-TZVPP counterpart. We assume this stabilization of the optical gap after the inclusion of diffuse functions in the basis-set to be spurious, in line with what was previously observed by Gui and co-workers.^33^ Also, because the addition of diffuse functions to the basis-set considerably reduces the good agreement with the experimentally measured UV–vis spectra for both [Cd(SPh)_4_]^2–^ and the [Cd(SPh)_4_]^2–^: 2 [N(CH_3_)_4_]^+^ ion pairs, discussed in more detail below.

The well-behaved nature and convergence with increasing basis-set size for the phenylthiolate-capped systems contrast with that for other anionic CdS systems. When the phenylthiolate ligands are replaced by bisulfide ligands, the differences in the optical gap predicted using the def2-SVP and def2-TZVPP basis-sets are smaller than that between the values obtained with the def2-TZVPP and def-QZVPP basis-sets, suggesting that the optical gap values are diverging rather than converging with increasing basis-set size (see Figure 3). The optical gap values of the methylthiolate-capped anionic systems appear to converge with increasing basis-set size, but the convergence is much slower than for the phenylthiolate capped particles. Worse yet, the change in the optical gap between the [Cd(SMe)_4_]^2–^ monomer and [Cd_4_(SMe)_10_]^2–^ decreases with increasing basis-set size, meaning that trends with particle size obtained with small basis-sets for these anionic particles might also be qualitatively wrong. Calculations with TD-DFT (TD-B3LYP) suggest that the same basis-set convergence issues also exist in the case of TD-DFT.

### Comparison with Experiment

**Figure 4:**
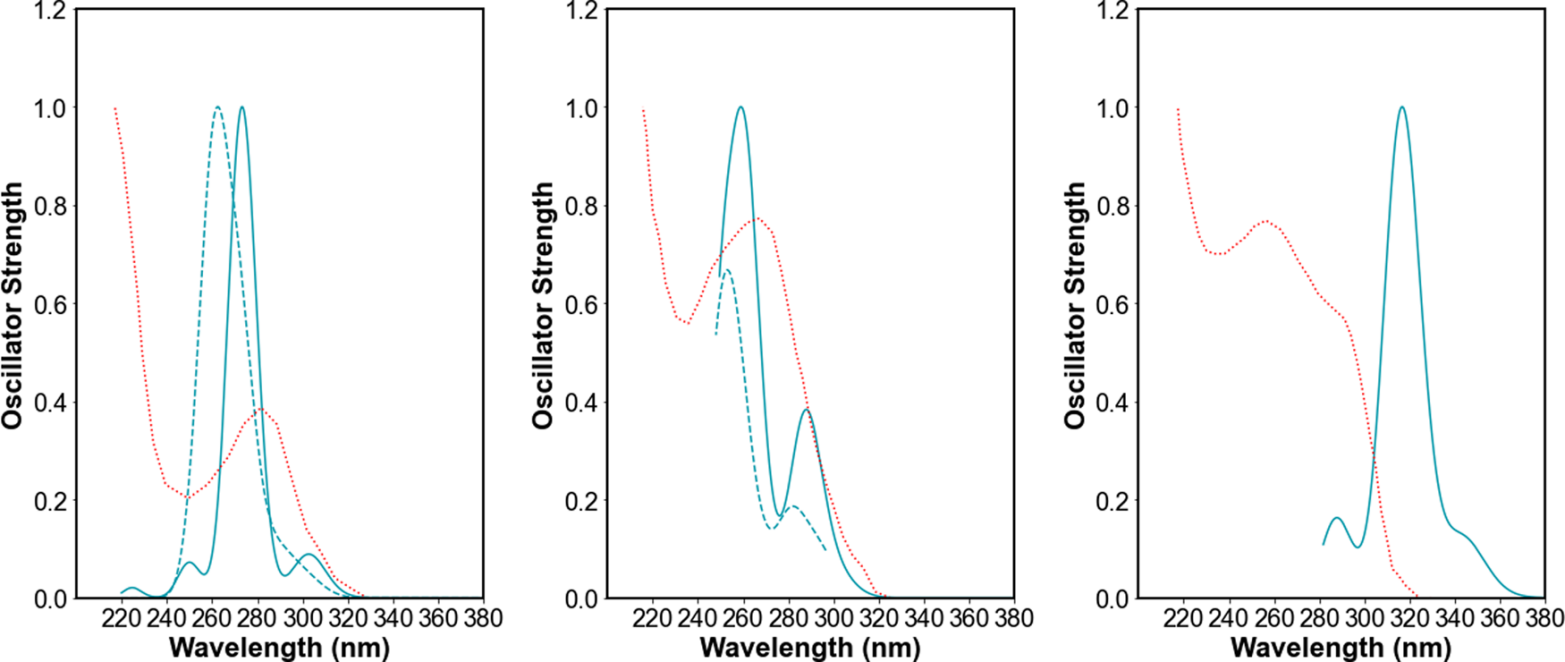
ev*GW*-BSE/def2-SVP predicted spectra of [Cd(SPh)_4_]^2–^ (left panel), [Cd_4_(SPh)_10_]^2–^ (middle panel), and [Cd_10_S_4_(SPh)_16_]^4–^ (right panel) in blue. The left and middle panels also include the predicted spectra of the corresponding ion pairs, [Cd(SPh)_4_]^2–^: 2 [N(CH_3_)_4_]^+^ and [Cd_4_(SPh)_10_]^2–^: 2 [N(CH_3_)_4_]^+^, respectively, as dashed lines. All panels moreover contain the corresponding experimental spectra extracted from ref (4) as a red dotted line. All spectra are normalized with respect to the highest (predicted) peak in the wavelength ranges shown.

Tuerk and co-workers reported spectra of solutions of the alkylammonium salts of [Cd(SPh)_4_]^2–^, [Cd_4_(SPh)_10_]^2–^, and [Cd_10_S_4_(SPh)_16_]^4–^ in dichloromethane.^4^ The results of the ev*GW*-BSE calculations agree well with the experimental spectra in terms of the position of the first excitation, with significant intensity probably responsible for the most red-shifted clear peak in the absorption spectra. For [Cd(SPh)_4_]^2–^, Tuerk and co-workers reported a peak at 282 nm (4.39 eV) and ev*GW*-BSE predicts a peak at 274 nm (4.52 eV) when using the def2-SVP basis-set (see Figure 4) and 282 nm (4.40 eV) when using the def2-TZVPP basis-set. Tuerk and co-workers report a peak at 275 nm (4.51 eV) for [Cd_4_(SPh)_10_]^2–^, and ev*GW*-BSE/def2-SVP predicts a peak at 262–259 nm (4.73–4.80 eV; see Figure 4). Finally, for [Cd_10_S_4_(SPh)_16_]^4–^, Tuerk and co-workers report a peak at 292 nm (4.25 eV) and ev*GW*-BSE/def2-SVP predicts a peak at 316 nm (3.91 eV; see Figure 4). For [Cd_10_S_4_(SPh)_16_]^4–^, we are limited in the number of excited states we can consider, so it is possible that if we could consider more states, the peak in the predicted spectra would lie closer to the experimental data. In the case of [Cd_4_(SPh)_10_]^2–^, ev*GW*-BSE/def2-SVP also predicts a shoulder at 288 nm (4.3 eV; see Figure 4), which is not clearly visible in the experimental spectrum but simultaneously not at odds with it. The predicted spectra of the ion pairs, see Figure 4, are consistent with those of the bare ions, bar a slight shift in the spectra. Finally, our calculations and experiment agree that the optical gap/absorption onset of [Cd_4_(SPh)_10_]^2–^ and [Cd_10_S_4_(SPh)_16_]^4–^ are much higher than that of bulk zincblende CdS, which has an experimental optical gap of 2.5 eV based on reflection spectroscopy measurements.^43^

### Character of the Excited States

**Figure 5:**
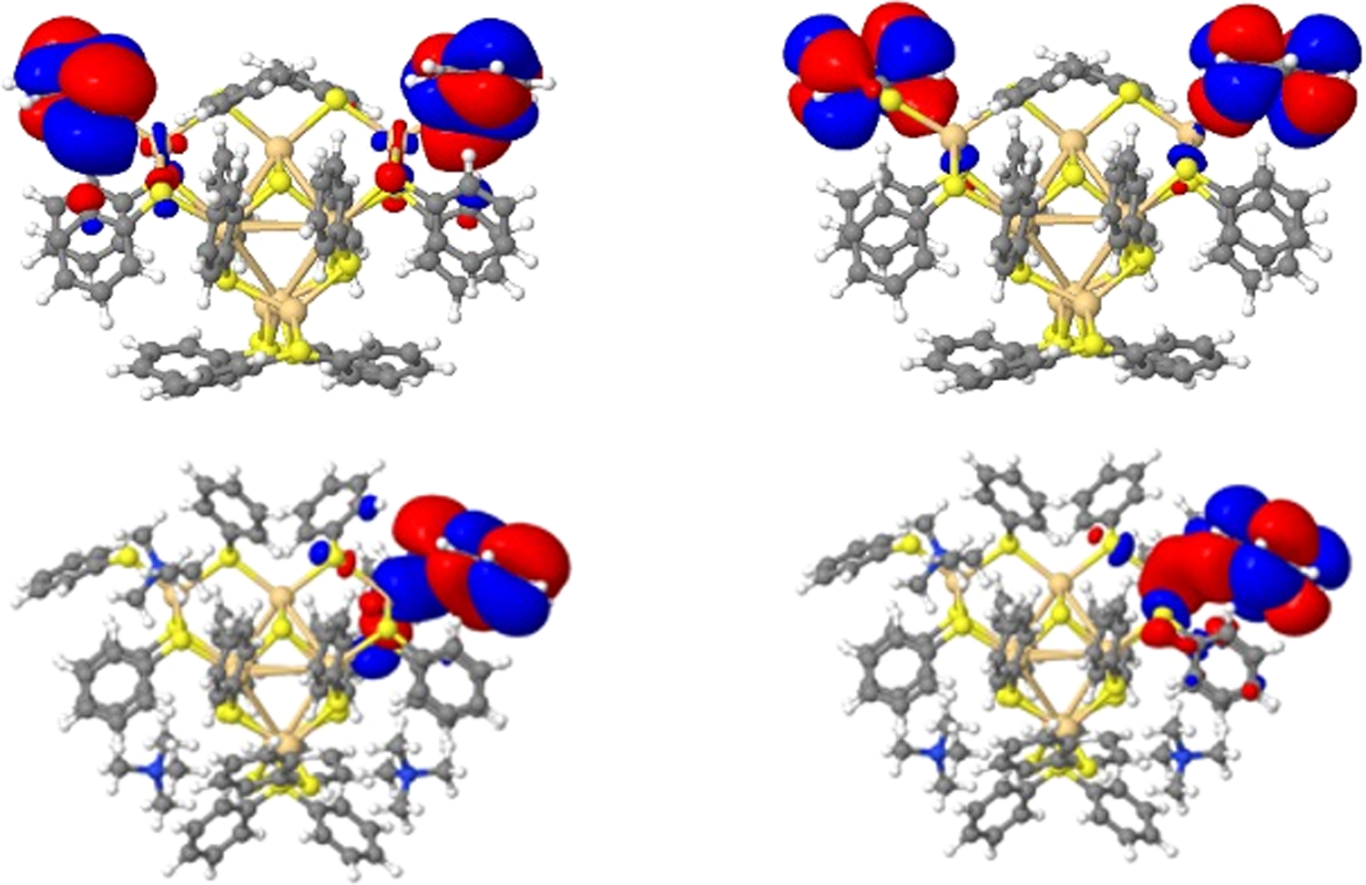
Leading natural transition orbitals for the hole (left) and excited electron (right) components of the lowest excited state for [Cd_10_S_4_(SPh)_16_]^4–^ (top row) and [Cd_10_S_4_(SPh)_16_]^4^: 4 [N(CH_3_)_4_]^+^ (bottom row).

Based on the natural transition orbitals, Figures 5 and S1, and the different contributions of the ligands, metal, and sulfur atoms to them, as analyzed by the TheoDORE code, each of the low-energy excited states of the phenylthiolate-capped particles is a mixture of two types of excitations. The first type of excitation is an intraligand excitation, where the hole is equally shared between the sulfur atoms and the phenyl rings of the four corner-capping ligands, while the electron localizes on just the phenyl rings of the same ligands, i.e., a ligand-based π + S(p) to π* (π → π* and n → π*) excitation. The other type of excitation is a ligand-to-metal (core) charge-transfer excitation, with the hole localized on the sulfur atoms of the corner-capping ligands and the electron delocalized over the cadmium atoms in the core. The exact mixture differs from excited state to excited state, but the intraligand excitation is always the dominant contribution to the mixture. The contribution of the sulfur atoms in the core of the particle, including those of the 3-coordinated sulfur atoms on the edges of the particles, is small to negligible in all cases. Higher energy, more blue-shifted excitations, like those responsible for the 275 nm absorption peak for [Cd_4_(SPh)_10_]^2–^, also appear to involve the 3-coordinated sulfur atoms and π-systems of the ligands on the edges of the particles.

Focusing on the lowest-energy excited state for each particle (the excited state responsible for the optical gap) for all particles studied here other than the [Cd_4_(SPh)_10_]^2–^: 2 [N(CH_3_)_4_]^+^ ion pair, this is almost a pure intraligand excitation. In contrast, each of the bright low-energy excited states, those discussed above in the context of the comparison to the experimental spectra other than the 275 nm absorption peak for [Cd_4_(SPh)_10_]^2–^, has a significant ligand-to-metal (core) charge-transfer contribution. The bright 275 nm absorption peak for [Cd_4_(SPh)_10_]^2–^ has, as discussed above, a more complicated character, involving all components of the particle.

The character of the excited state predicted by the ev*GW*-BSE calculations agrees with the interpretation of the experimental spectra by Tuerk and co-workers, who write “*the absorption spectra of the Cd*_*n*_*clusters are most likely a composite of intraligand and ligand-to-metal charge-transfer (LMCT) transitions”*.^4^

Comparing the optical gap for each of the studied phenylthiolate-capped particles with the corresponding predicted fundamental gap value, the excited state corresponding to the optical gap for each particle is clearly excitonic in character with exciton binding energies of 3 eV and larger. Moreover, all low-energy optical excitations, such as those discussed when comparing the experimentally measured absorption spectra, have excitation energies below the fundamental gap and are also excitonic in character. We are not aware of measurements or predictions of the exciton binding energy in bulk zincblende CdS, but the experimentally reported bulk exciton binding energy for the wurtzite polymorph of CdS is 29 meV (0.029 eV).^44^ Hence, the excitons in the phenylthiolate-capped CdS particles are likely bound 2 orders of magnitude stronger than in the bulk.

Finally, as can be seen in Figure S2 in the Supporting Information, the character of the highest occupied and especially the lowest unoccupied quasiparticle states when analyzed in terms of the underlying Kohn–Sham states are qualitatively different from the corresponding natural transition orbitals. The lowest unoccupied quasiparticle states are essentially delocalized over the particles. This difference between the character of the quasiparticle and the optically excited states is evidence again of the strong excitonic character of the latter for these particles.

### Change with Particle Size

**Figure 6:**
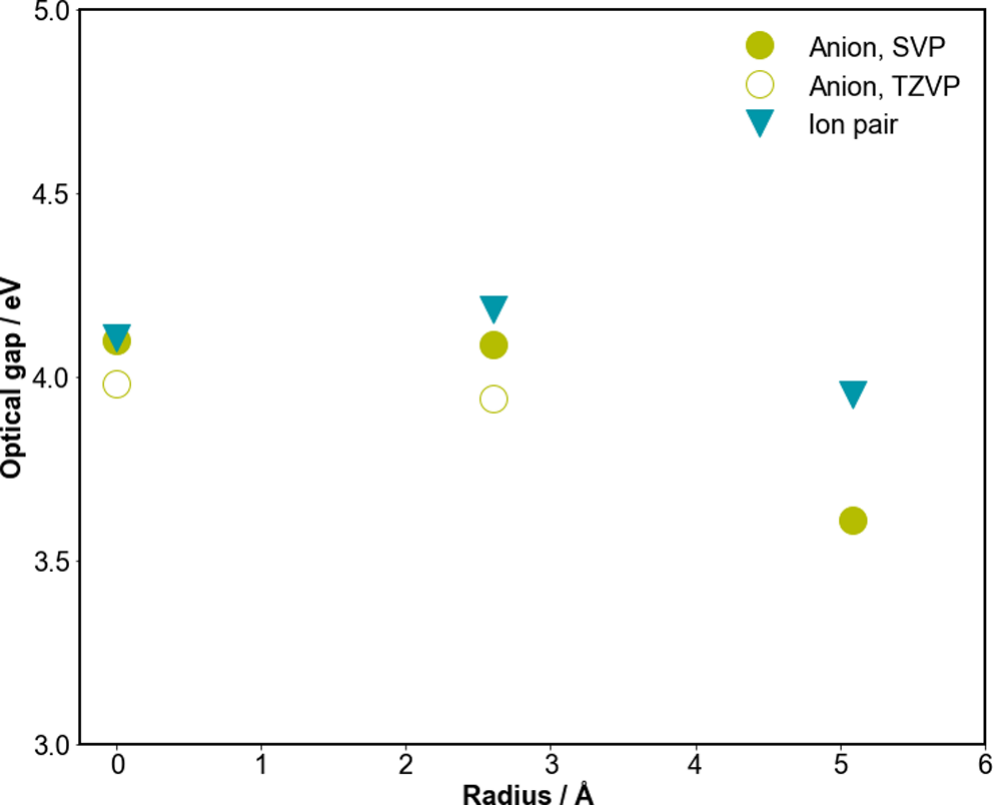
ev*GW*-BSE predicted the optical gap versus the radius of the inorganic core of the anionic particles, as well as the corresponding ion pairs.

Focusing on [Cd_4_(SPh)_10_]^2–^ and [Cd_10_S_4_(SPh)_16_]^4–^, as can be seen in Figure 6, the optical gap of both the anionic particles and the corresponding ion pairs increases with a decreasing radius of the inorganic core of the particle and thus the particle size (by 0.48 and 0.23 eV, respectively). Similarly, the lowest bright excited state of [Cd_4_(SPh)_10_]^2–^ and [Cd_10_S_4_(SPh)_16_]^4–^ shifts to higher energy with a decreasing core radius and particle size (by 0.3–0.9 eV depending on which peaks one uses). The optical gap and lowest bright excited state of the ion pairs display the same trend. It is tempting to explain this blue shift of both the optical gap and lowest bright excited state in terms of quantum confinement, especially as noted above; bulk zincblende CdS has a much smaller optical gap than the phenylthiolate-capped particles. Indeed, it stands to reason that the energy of bright states should be sensitive to particle size because of the ligand-to-metal (core) charge-transfer contribution. However, this cannot be the (whole) explanation, as the excited state responsible for the optical gap for most systems is, as noted above, a pure intraligand excitation. In the absence of significant changes in (de)localization, we wonder if the blue shift in the optical gap is actually due to a Stark-like shift. Such a Stark-like shift would be related to the charge of the core, including the ligands on the edges of the particle but excluding the four corner-capping ligands, changing from +2 to 0 when going from [Cd_4_(SPh)_10_]^2–^ (net particle charge −2, combined charge on the four corner-capping ligands −4, core charge +2) to [Cd_10_S_4_(SPh)_16_]^4–^ (net particle charge −4, combined charge on the four corner-capping ligands −4, core charge 0). This change in the charge of the core then changes the electric field experienced by the corner-capping ligand, responsible for the intraligand character. Such an explanation would be in line with the fact that the charge on the core of [Cd_4_(SPh)_10_]^2–^ and [Cd(SPh)_4_]^2–^ is the same and their optical gaps are similar, see Figure 6, and the fact that the fundamental gap, which have ligand-to-metal (core) charge transfer instead of intraligand character, in contrast steadily decreases with increasing core size (see Figure S3 in the SI).

The optical gap of the ion pairs, finally, displays, as can be seen in Figure 6, a similar trend as the anionic particles. This is in line with the fact that while the ion pairs are neutral, the electric field resulting from the charges on the inorganic core, and now also the alkylamine counterions, persists in such ionically bonded complexes.

## Discussion

To reduce the computational cost of the calculations, phenylthiolate ligands are often simplified to methylthiolate or bisulfide ligands. The results above show that this is a potentially questionable approach. First, the phenylthiolate ligands are clearly not electronically innocent, and calculations on methylthiolate- or bisulfide-capped nanoparticles are not going to reproduce the intraligand or ligand-to-metal (core) excitations of the phenylthiolate-capped particles. Perhaps, even more concerning are the basis-set convergence issues, although that is probably only a problem for charged particles, like those studied here, and not for neutral particles.

In terms of the (de)localization of the excited state, the phenylthiolate-capped particles studied here clearly do not behave like the textbook cartoon description of quantum confinement as the excited states are fully or at least partly localized on the ligands. The particles do display a blue shift of the optical properties with decreasing particle size, and in the case of the bright absorption peaks observed experimentally, this blue shift might be explicitly linked to the increase in size via the excited electron part of the ligand-to-metal (core) charge-transfer excitation component, which is delocalized over the metal atoms in the particle core. However, this is far from a bulk-like excited state in which both the hole and excited electron components are delocalized over the inorganic core of the particle and the ligand innocent spectators. As such, the phenylthiolate-capped CdS particles prepared experimentally as atomically precise nanoparticles and studied here theoretically are very different from bulk CdS other than in terms of the atomic structure of the inorganic core. This is in contrast to, for example, hydrogenated silicon, which we studied previously,^25^ which much more closely resembles bulk silicon.

Nanoparticles generally have a larger exciton binding energy than the bulk due to the reduced dielectric screening in nanoparticles.^25^ However, in the case of the phenylthiolate-capped CdS nanoparticles, the larger exciton binding energy is also, at least partly, a result of the fact that the character of the excited states in the nanoparticles is fundamentally different from those in the bulk.

We are limited in terms of the size of the particles we can study computationally. However, it stands to reason that the character of the excited state in the larger phenylthiolate particles, for example, those prepared by Herron and co-workers,^3^ whose optical properties experimentally are a smooth extension of those studied here, will be similar and equally far from the textbook cartoon description of quantum confinement. It is possible that when the core of the particles becomes big enough, intracore bulk-like excitations become lower in energy than the ligand-to-metal and intraligand excitations, and the ligand-capped CdS particles will start behaving like the textbook picture. However, it is difficult to predict if and at what size such a transition would take place based on our data. We see no evidence of such intracore excitations for the particle studied here in the excitation energy range studied.

Similarly, while we cannot currently predict fluorescence spectra with *GW*-BSE, the fact that we predict that the low-energy excited states in phenylthiolate-capped particles involve ligands agrees with the same observation by Herron and co-workers for the experimental fluorescence spectra.^3^ As noted in the Introduction section, Tuerk and co-workers^4^ propose that fluorescence occurs from a ligand-to-metal charge-transfer state. While we generally predict that the lowest excited state is a pure intraligand state, it is not unreasonable to suppose that upon relaxation of the excited state, the relative intraligand and ligand–metal charge-transfer contributions will change and that a relaxed excited state has a larger ligand–metal charge-transfer contribution than at the ground-state geometry.

From a synthetic perspective, if one wants to synthesize CdS particles where both the hole and excited electron components are delocalized over the inorganic core of the particles, the use of phenylthiolate and related aromatic ligands clearly appears challenging. However, the calculations on the methylthiolate- and bisulfide-terminated particles, while clearly problematic for the reasons discussed above, suggest that even for nonaromatic ligands it might be hard to achieve an excitation not involving the ligands. While with such ligands there are no intraligand excitations, the lowest excitations appear to all have a ligand-to-metal (core) charge-transfer character involving the sulfur atoms of the corner-capping ligands.

## Conclusions

The results of ev*GW*-BSE calculations on phenylthiolate-capped nanoparticles agree well with the absorption spectra measured experimentally for these particles. Analysis of the character of the underlying excited states shows that the low-energy excited states for such particles are a mixture of π → π* and n → π*, intraligand and ligand-to-metal charge-transfer excitations, where the former are always the dominant contribution. There are no low-energy excited states for which both the hole and the excited electron components are predominantly delocalized over the inorganic core of the particles (bulk-like excitons) as might have been naively expected to exist. In line with the experiment, the optical gap values and overall spectra of the particles are predicted to blue-shift with decreasing particle size, but the mechanism is far from the cartoon textbook description of quantum confinement. Specifically, the mechanism of the blue shift most likely combines a clamping of the excited electron–hole component of the ligand-to-metal charge-transfer contribution and a Stark effect for the intraligand contribution. Overall, the phenylthiolate-capped CdS particles prepared experimentally as atomically precise nanoclusters and studied here theoretically are very different from bulk CdS other than in terms of the atomic structure of the inorganic core.

The important role of the ligands in the excitations means that calculations that simplify the phenylthiolate ligands to methylthiolate or bisulfide cannot qualitatively reproduce the experimental spectrum. Worse still, calculations with such a simplified ligand display basis-set convergence issues.

## Supporting Information Available

Tables of the predicted fundamental and optical gap values (PDF)
DFT optimized structures of all relevant particles (ZIP)
