# Supplementary material for: Optical Properties of Phenylthiolate-Capped CdS Nanoparticles
Source: J Phys Chem C Nanomater Interfaces. 2025 Jan 10;129(3):1797–805. doi: 10.1021/acs.jpcc.4c06753 (PMC11770759; doi:10.1021/acs.jpcc.4c06753)
Supplement: Supplementary file 1 — jp4c06753_si_001.pdf [file jp4c06753_si_001.pdf]

Electronic Supporting Information

Optical Properties of Phenylthiolate Capped CdS Nanoparticles

Eimear Madden, Martijn A. Zwijnenburg*

Department of Chemistry, University College London, 20 Gordon Street,

London WC1H 0AJ, UK.

Table S1 *G_0W_0-BSE predicted fundamental gap and optical gap values of $[Cd(SPh)_4]^{2-}$, $[Cd_4(SPh)_{10}]^{2-}$ and $[Cd_{10}S_4(SPh)_{16}]^{4-}$ calculated with the def2-SVP basis-sets. All values in eV.*

	def2-SVP	
	Δ_F	Δ_O
$[Cd(SPh)_4]^{2-}$	7.49	3.47
$[Cd_4(SPh)_{10}]^{2-}$	6.73	3.54
$[Cd_{10}S_4(SPh)_{16}]^{4-}$	6.61	3.12

Table S2 qs*GW-BSE predicted fundamental gap and optical gap values of $[Cd(SPh)_4]^{2-}$ - calculated with the def2-SVP basis-sets. All values in eV.*

	def2-SVP	
	Δ_F	Δ_O
$[Cd(SPh)_4]^{2-}$	8.20	4.19

Table S3 *evGW-BSE predicted fundamental gap and optical gap values of $[Cd(SPh)_4]^{2-}$ and $[Cd_4(SPh)_{10}]^{2-}$ calculated with the def2-SVP, def2-TZVPP and def2-QZVPP basis-sets. All values in eV.*

	def2-SVP		def2-TZVPP		def2-QZVPP	
	Δ_F	Δ_O	Δ_F	Δ_O	Δ_F	Δ_O
$[Cd(SPh)_4]^{2-}$	8.12	4.10	7.86	3.98	7.77	3.95
$[Cd_4(SPh)_{10}]^{2-}$	7.31	4.09	7.09	3.94		

Table S4 *evGW-BSE predicted fundamental gap and optical gap values of the different ion pairs- calculated with the def2-SVP and def2-TZVPP basis-sets. All values in eV.*

	def2-SVP		def2-TZVPP	
	Δ_F	Δ_O	Δ_F	Δ_O
$[Cd(SPh)_4]^{2-}$: 2 $[TMA]^+$	7.86	4.11	7.59	3.97
$[Cd_4(SPh)_{10}]^{2-}$: 2 $[TMA]^+$	6.88	4.18		
$[Cd_{10}S_4(SPh)_{16}]^{4-}$: 4 $[TMA]^+$	6.96	3.95		

Table S5 *evGW-BSE predicted fundamental gap and optical gap values of $[Cd(SPh)_4]^{2-}$ calculated with the def2-SVPD, def2-TZVPPD and def2-QZVPPD basis-sets. Calculations performed as single-point calculations on geometries optimised with the def2-SVP, def2-TZVPP and def2-QZVPP basis-sets, respectively. All values in eV.*

	def2-SVPD		def2-TZVPPD		def2-QZVPPD	
	Δ_F	Δ_O	Δ_F	Δ_O	Δ_F	Δ_O
$[Cd(SPh)_4]^{2-}$	7.76	3.89	6.63	3.34	6.40	3.36

Table S6 *evGW-BSE predicted fundamental gap and optical gap values of* $[Cd_{10}S_4(SPh)_{16}]^{4-}$ *calculated with the def2-SVP basis-sets. All values in eV.*

	def2-SVP	
	Δ_F	Δ_O
$[Cd_{10}S_4(SPh)_{16}]^{4-}$	7.12	3.61

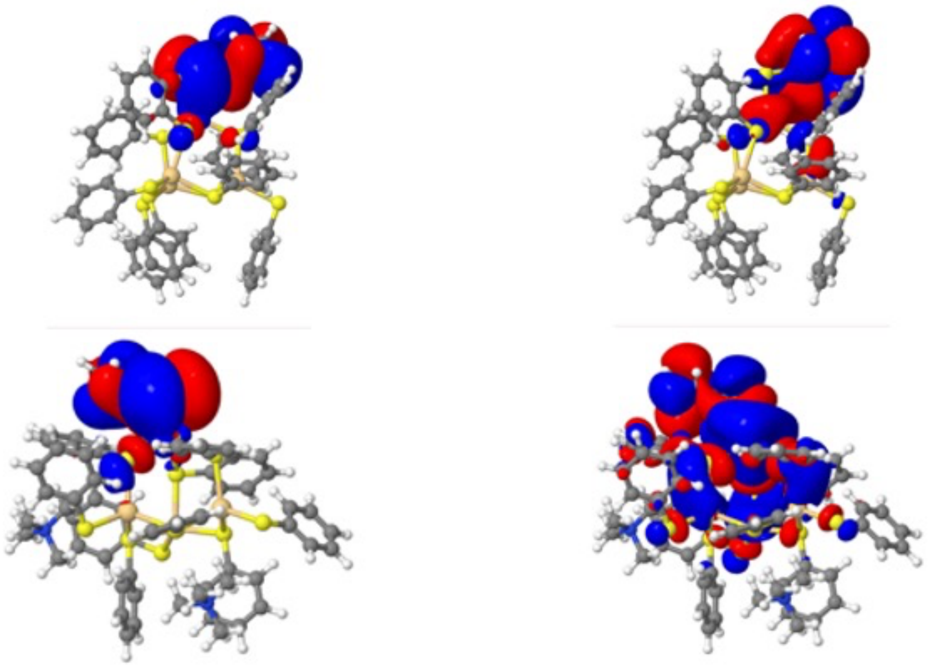

Fig. S1 *Leading natural transition orbitals for the hole (left) and excited electron (right) component of the lowest excited state for* $[Cd_4(SPh)_{10}]^{2-}$ *(top row) and* $[Cd_4(SPh)_{10}]^{2}$*: 2* $N(CH_3)_4]^{+}$ *(bottom row).*

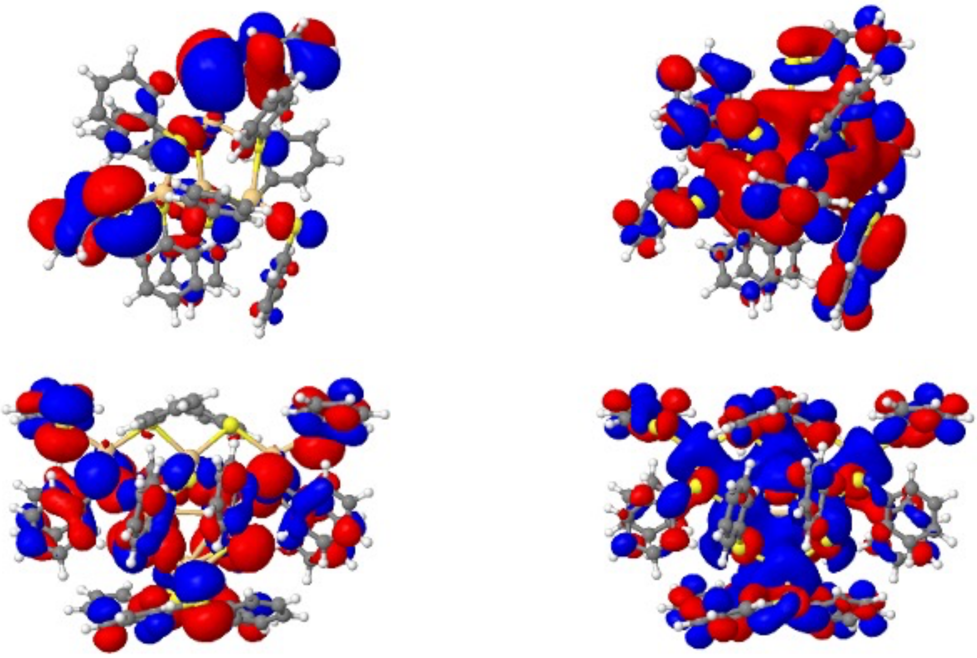

Fig. S2 *Highest occupied (left) and lowest unoccupied (right) Kohn-Sham orbitals of* $[Cd_4(SPh)_{10}]^{2-}$ *(top row) and* $[Cd_{10}S_4(SPh)_{16}]^{4-}$ *(bottom row).*

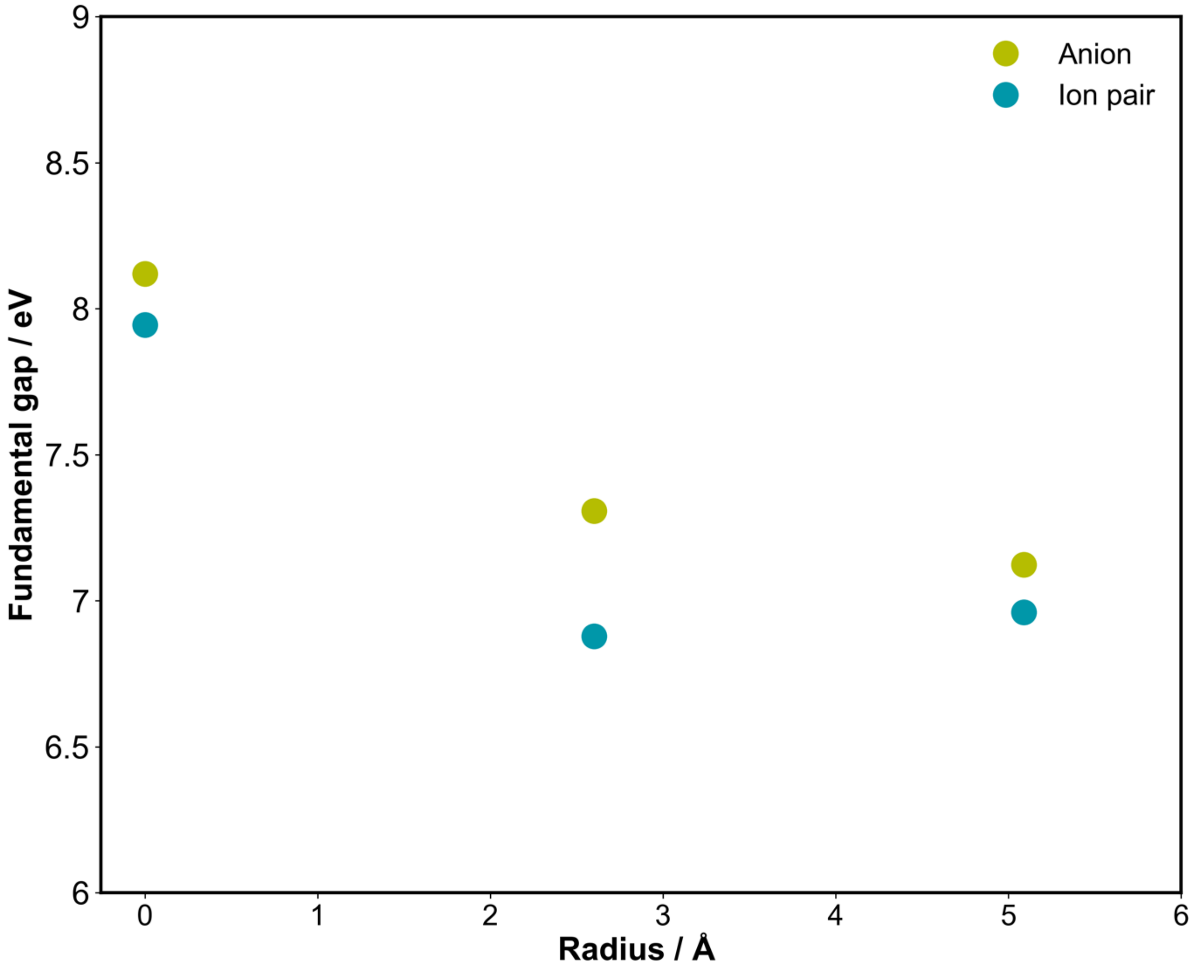

Fig. S3 *evGW predicted fundamental gap versus the radius of the inorganic core of the anionic particles*
